# Geometric Calibration and Radiometric Correction of LiDAR Data and Their Impact on the Quality of Derived Products

**DOI:** 10.3390/s110909069

**Published:** 2011-09-21

**Authors:** Ayman F. Habib, Ana P. Kersting, Ahmed Shaker, Wai-Yeung Yan

**Affiliations:** 1 Department of Geomatics Engineering, University of Calgary, 2500 University Drive NW, Calgary, Alberta T2N 1N4, Canada; E-Mail: ana.kersting@ucalgary.ca; 2 Department of Civil Engineering, Ryerson University, 350 Victoria Street, Toronto, Ontario M5B 2K3, Canada; E-Mails: ahmed.shaker@ryerson.ca (A.S.); waiyeung.yan@ryerson.ca (W.-Y.Y.)

**Keywords:** LiDAR, geometric calibration, radiometric correction

## Abstract

LiDAR (Light Detection And Ranging) systems are capable of providing 3D positional and spectral information (in the utilized spectrum range) of the mapped surface. Due to systematic errors in the system parameters and measurements, LiDAR systems require geometric calibration and radiometric correction of the intensity data in order to maximize the benefit from the collected positional and spectral information. This paper presents a practical approach for the geometric calibration of LiDAR systems and radiometric correction of collected intensity data while investigating their impact on the quality of the derived products. The proposed approach includes the use of a quasi-rigorous geometric calibration and the radar equation for the radiometric correction of intensity data. The proposed quasi-rigorous calibration procedure requires time-tagged point cloud and trajectory position data, which are available to most of the data users. The paper presents a methodology for evaluating the impact of the geometric calibration on the relative and absolute accuracy of the LiDAR point cloud. Furthermore, the impact of the geometric calibration and radiometric correction on land cover classification accuracy is investigated. The feasibility of the proposed methods and their impact on the derived products are demonstrated through experimental results using real data.

## 1. Introduction

The importance of geometric calibration and radiometric correction of active remote sensing data has been emphasized for Japan Earth Resources Satellite-1 Synthetic Aperture Radar (JERS-1 SAR) [1], RADARSAT [2], European Remote-Sensing Satellite Synthetic Aperture Radar (ERS SAR) [3], and Advanced Land Observation Satellite Phased Array type L-band Synthetic Aperture Radar (ALOS PALSAR) [4]. Different rigorous geometric and radiometric correction models were developed by considering the scanning geometry, backscattering mechanism, and terrain induced distortions [5]. It has been shown that these factors have significant influence on the derived data products. LiDAR systems have similar operational principles to RADAR and SAR systems. Laser energy is emitted and the backscattered energy from the object space is recorded by the LiDAR system. The backscattered energy and the time delay between the signal emission and reception are used to derive a 3D point cloud, which is represented by the *XYZ* coordinates and the intensity *I* representing the peak backscattered laser energy from the object. Due to systematic errors in the LiDAR system parameters and measurements, geometric calibration and radiometric correction of the positional and intensity data are essential to ensure the best accuracy of the delivered products.

The geometric calibration of LiDAR systems aims at estimating and removing all the systematic errors from the point cloud coordinates such that only random errors are left. Systematic errors in the LiDAR data are mainly caused by biases in the system parameters, e.g., biases in the mounting parameters relating the system components (lever arm and boresight angles) and biases in the measured ranges and mirror angles. The elimination/reduction of such systematic errors or their impact has been the focus of the LiDAR research community in the past few years. The existing approaches can be classified into two main categories: system-driven (calibration) and data-driven (strip adjustment) procedures. This categorization is mainly related to the nature of the utilized data and mathematical model. System-driven (calibration) procedures are based on the physical sensor model relating the system measurements/parameters to the ground coordinates of the LiDAR points. These procedures incorporate the system’s raw data or at least the trajectory and time-tagged point cloud for the estimation of biases in the system parameters with the help of the LiDAR point positioning equation. In this paper, the term “raw data” is used to denote all the quantities present in the LiDAR point positioning equation (*i.e.*, position and orientation information as well as the measured range and scan angle for each pulse). Moreover, the utilized sequence of rotation angles, which define the system attitude and boresight angles, have to be clearly specified. The access to the system raw measurements is usually restricted to LiDAR system manufacturers. Such a restriction has triggered the development of methods that only utilize the *XYZ* coordinates of the LiDAR point cloud. These methods are classified as data-driven (or strip adjustment) procedures [6–9]. The major drawback of such methods is the mathematical model employed to relate the LiDAR strips and the reference frame. The effects of systematic errors in the system parameters are usually modeled by an arbitrary transformation function between the laser strip and the reference frame coordinate systems. Depending on the nature of the inherent biases in the LiDAR system parameters, the utilized transformation function might not be appropriate.

As already mentioned, rigorous geometric calibration procedures are based on the physical mathematical model (*i.e.*, LiDAR point positioning equation) that involves the raw data [10–12] or at least the trajectory and time-tagged point cloud [13–15]. In spite of the relaxed data requirements in [13–15], these methods have some shortcomings. For instance, in [13] and [14], only biases in the boresight angles are estimated during the calibration procedure. Moreover, the parameters describing the utilized surface in [13] are estimated within the calibration procedure. Thus, the number of estimated parameters depends on the extent of the area being utilized in the calibration procedure. The introduced procedure in [14] estimates the boresight angles using observed discrepancies between conjugate surface elements in overlapping LiDAR strips. The discrepancies are obtained through a matching procedure that uses interpolated data. The implemented matching procedure does not lead to accurate estimates of the horizontal discrepancies when compared to the estimated vertical discrepancy. Therefore, the estimated biases in the boresight pitch and heading angles are of lower accuracy than the boresight roll angle since the former lead to horizontal discrepancies while the latter leads to horizontal as well as vertical discrepancies among overlapping strips. In [15], point primitives are utilized to establish the correspondence between overlapping strips. Due to the irregular nature of the LiDAR points, the identification of distinct and conjugate points in overlapping strips is quite difficult and not reliable. Moreover, this method assumes that the true ground coordinates can be derived by averaging the coordinates of tie points in overlapping strips. The averaging process will not lead to the correct estimate of the LiDAR point in the presence of some systematic errors. Finally, the above studies did not elaborate on the required flight configuration and control requirement for a reliable and economic calibration technique.

Radiometric correction of the LiDAR data is a relatively new research area. It aims at converting the recorded intensity data into the spectral reflectance of an object. The radar equation has been proposed for radiometric correction of the LiDAR intensity data in [16] and [17]. The radar equation considers the effects of the measured laser range, angle of reflection, and atmospheric attenuation to retrieve the surface reflectance in the near infrared red (NIR) spectrum. Radiometric calibration of LiDAR data is proposed by researchers at the Finnish Geodetic Institute in a number of recent publications [18–20]. This work is based on the utilization of reference targets (e.g., Spectralon reference plates) and ground features (e.g., beach sand and asphalt) to calibrate the LiDAR intensity data by comparing the measured reflectance with *in situ* measurements from spectroradiometer and NIR digital camera. The study has established that the homogeneity and the size of the calibration targets play a critical role in the radiometric calibration. Effects of the system parameters such as the aperture size [21] and the automatic gain control [22] have been also investigated. Practical methods were developed to eliminate the effects of these parameters through absolute correction and relative calibration approaches.

Despite the previous research on geometric calibration of LiDAR systems and radiometric correction of the intensity data, the impact of these approaches on the quality of the derived products is still an open research area. This paper presents methods for geometric calibration and radiometric correction of airborne LiDAR data while evaluating their effect on the geo-positional accuracy and classification of the intensity data. The geometric calibration involves a quasi-rigorous procedure for the estimation of biases in the system parameters. The geo-positioning accuracy of the adjusted point cloud is assessed based on quantifying the degree of compatibility between LiDAR and control surfaces before and after the calibration process. The radiometric correction utilizes the radar equation to determine the spectral reflectance of objects. Then, land cover classification is conducted. Accuracy assessment with checkpoints acquired from an orthophoto is used to assess and compare the classification results from the original and the geometrically calibrated and radiometrically corrected datasets.

## 2. Geometric System Calibration

In this section, the proposed geometric calibration procedure for the estimation of biases in the system parameters is described. This method, which is denoted as “Quasi-Rigorous” since it only requires the time-tagged point cloud and trajectory position data, utilizes LiDAR data in overlapping strips. Biases in the system parameters are estimated by reducing discrepancies between conjugate surface elements in overlapping strips and control data, if available.

The proposed method will be explained in the following subsections. First, the mathematical model relating conjugate surface elements in overlapping LiDAR strips as well as overlapping LiDAR and control surfaces in the presence of systematic errors is derived. Based on the analysis of the derived mathematical model, remarks regarding the necessary flight and control configuration for LiDAR system calibration are outlined. The established mathematical model for the calibration procedure is derived based on point primitives (*i.e.*, conjugate points in overlapping point cloud data). However, point-to-point correspondence cannot be assumed due to the irregular/semi-random nature of the LiDAR points. In this work, conjugate point and Triangular Irregular Network (TIN) patch pairs are proposed as primitives. A modification to the traditional Gauss Markov Stochastic model that would allow for the utilization of these conjugate primitives while using the established point-based mathematical model is finally described.

### 2.1. Mathematical Model

**Figure 1.**
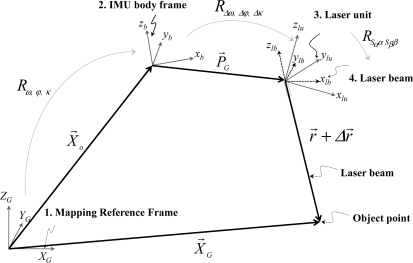
Coordinate systems and involved quantities in the LiDAR point positioning equation.

The coordinates of the LiDAR points are the result of combining the derived measurements from each of its system components, as well as the mounting parameters relating such components. The relationship between the system measurements and parameters is embodied in the LiDAR point positioning equation [23–24], Equation (1). The position of an object point *X⃗*_G_ is derived through the summation of three vectors (*X⃗_o_*, *P⃗_G_*, and *r⃗*) after applying the appropriate rotations (*R*_*ω,φ,κ*_, *R*_*Δω,Δφ,Δκ*_, and *R*_*S_α_αS_β_β*_ (refer to Figure 1). In this equation, *X⃗_o_* is the vector from the origin of the ground reference frame to the origin of the Inertial Measurement Unit (IMU) coordinate system, *P⃗_G_* (lever arm vector) is the offset between the IMU and laser unit coordinate systems (w.r.t. the IMU body frame), and *r⃗* is the laser range vector whose magnitude is equivalent to the distance from the laser firing point to its footprint. It should be noted that *X⃗_o_* is derived through the Global Positioning System/Inertial Navigation System (GPS/INS) integration process while considering the lever arm vector between the IMU body frame and the phase centre of the GPS antenna. The term *R*_*ω,φ,κ*_ stands for the rotation matrix relating the ground and IMU coordinate systems—which is derived through the GPS/INS integration process. The term *R_Δω,Δφ,Δκ_* represents the rotation matrix relating the IMU and laser unit coordinate systems—which is defined by the boresight pitch (*Δω*), roll (*Δφ*), and heading (*Δκ*) angles (considering the y-axis of the IMU body frame aligned along the flight direction), while the term *R*_*S_α_αS_β_β*_ refers to the rotation matrix relating the laser unit and laser beam coordinate systems with *α* and *β* being the mirror scan angles. *S_α_* and *S_β_* are the scale factors of the angles measured by the scanner, while *Δr* is a systematic error in the measured range. The involved quantities in the LiDAR point positioning equation are all measured during the acquisition process except for the mounting parameters (*i.e.*, the lever arm and boresight angles), the scan angles scale factors, and the range error; these parameters are usually determined through a calibration procedure.

(1) $${\vec{X}}_{G}={\vec{X}}_{o}+R_{\omega ,\varphi ,\kappa }{\vec{P}}_{G}+R_{\omega ,\varphi ,\kappa }R_{\Delta \omega ,\Delta \varphi ,\Delta \kappa }R_{S_{\alpha }\alpha \;S_{\beta }\beta }\;\left[\begin{array}{c} 0\\ 0\\ -\left(r+\Delta r\right) \end{array}\right]$$

(2) $$\begin{array}{c} {\vec{X}}_{G}={\vec{X}}_{o}+\left[\begin{array}{ccc} \cos\kappa & -\sin k & 0\\ \sin k & \cos\;\kappa & 0\\ 0 & 0 & 1 \end{array}\right]\;\left[\begin{array}{c} \Delta X\\ \Delta Y\\ \Delta Z \end{array}\right]+\left[\begin{array}{ccc} \cos\kappa & -\sin k & 0\\ \sin k & \cos\kappa & 0\\ 0 & 0 & 1 \end{array}\right]\;\left[\begin{array}{ccc} 1 & -\Delta \kappa & \Delta \varphi \\ \Delta \kappa & 1 & -\Delta \omega \\ -\Delta \varphi & \Delta \omega & 1 \end{array}\right]\;\left[\begin{array}{c} -\left(r+\Delta r\right)\sin\left(S\beta \right)\\ 0\\ -\left(r+\Delta r\right)\cos\left(S\beta \right) \end{array}\right]\\ ={\vec{X}}_{o}+\left[\begin{array}{ccc} \cos\kappa & -\sin k & 0\\ \sin k & \cos\;\kappa & 0\\ 0 & 0 & 1 \end{array}\right]\;\left[\begin{array}{c} \Delta X\\ \Delta Y\\ \Delta Z \end{array}\right]+\left[\begin{array}{ccc} \cos\kappa & -\sin k & 0\\ \sin k & \cos\kappa & 0\\ 0 & 0 & 1 \end{array}\right]\;\left[\begin{array}{ccc} 1 & -\Delta \kappa & \Delta \varphi \\ \Delta \kappa & 1 & -\Delta \omega \\ -\Delta \varphi & \Delta \omega & 1 \end{array}\right]\;\left[\begin{array}{c} x\\ 0\\ z \end{array}\right] \end{array}$$

where

– *ΔX, ΔY, ΔZ* are the components of the lever arm vector *P⃗_G_*,
– *z* is the vertical coordinate of the object point with respect to the laser unit coordinate system,
– *x* is the lateral coordinate of the object point with respect to the laser unit coordinate system, which is the lateral distance (with the appropriate sign) between the LiDAR point in question and the projection of the flight trajectory onto the ground.

The LiDAR point positioning mathematical model presented in Equation (1) can be simplified to the form in Equation (2) after considering the following assumptions: linear scanner, vertical system, and small boresight angles. For a linear scanner, the mirror is rotated in one direction only, leading to zero values for *α* and *S_α_*. To simplify the utilized symbols, the scale factor *S_β_* will be represented here forth by *S*. One should note that in Equation (2), we consider the convention of right-forward-up (X-Y-Z) coordinate systems for the laser unit and IMU body frame with the Y-axis aligned along the flight direction. The deterministic LiDAR equations for point positioning (Equations (1) and (2)) can be represented in a symbolic form by Equation (3). This equation indicates that the true coordinates of a given point *X⃗_G_* (*True*) is derived using the true values for the system parameters *x_t_* and noise free measurements *l_nf_*. However, if we only have access to biased system parameters *x_b_* as well as noisy measurements *l_n_*, the bias/noise contaminated coordinates of a given point *X⃗_G_*(*b/n*) can be expressed by Equation (4). The expression in Equation (3) can be rewritten in the form in Equation (5), where *δx* denotes the biases in the system parameters and *e* denotes the noise in the measurements:

(3) $${\vec{X}}_{G}\left(\mathrm{True}\right)=f\left(x_{t},l_{\mathrm{nf}}\right)$$

(4) $${\vec{X}}_{G}\left(b/n\right)=f\left(x_{b},l_{n}\right)$$

(5) $${\vec{X}}_{G}\left(\mathrm{True}\right)=f\left(x_{b}-\delta x,l_{n}-e\right)$$

Equation (5) can be expanded using Taylor’s series to the form in Equation (6) after ignoring second and higher order terms. This equation can be rewritten according to the form in Equation (7), which indicates that the bias/noise contaminated coordinates *X⃗_G_*(*b/n*) is equivalent to the summation of the true coordinates *X⃗_G_*(*True*) and other terms that describe the impact of biases in the system parameters and noise in the system measurements. Starting from Equation (2), the mathematical expression for the impact of biases in the system parameters on the derived ground coordinates *δX⃗_G_* is given in Equation (8):

(6) $${\vec{X}}_{G}\left(\mathrm{True}\right)\approx f\left(x_{b},l_{n}\right)-\partial f/\partial x{|}_{x_{b},l_{n}}\delta x-\partial f/\partial l{|}_{x_{b},l_{n}}e={\vec{X}}_{G}\left(b/n\right)-A\delta x-\mathrm{Be}$$

(7) $${\vec{X}}_{G}\left(b/n\right)={\vec{X}}_{G}\left(\mathrm{True}\right)+A\delta x+\mathrm{Be}={\vec{X}}_{G}\left(\mathrm{True}\right)+\delta {\vec{X}}_{G}+\mathrm{Be}$$

(8) $$\begin{array}{c} \delta {\vec{X}}_{G}=\left[\begin{array}{c} \cos\kappa \;\delta \Delta X-\sin k\;\delta \Delta Y+\sin\kappa \;z\;\delta \Delta \omega +\cos\kappa \;z\;\delta \Delta \varphi \\ \sin\kappa \;\delta \Delta X+\;\cos k\;\delta \Delta Y-\cos\kappa \;z\;\delta \Delta \omega +\sin\kappa \;z\;\delta \Delta \varphi \\ \delta \Delta Z-x\;\delta \Delta \varphi \end{array}\right]\\ +\left[\begin{array}{c} -\sin\kappa \;x\;\delta \Delta \kappa -\cos k\;\sin\left(S\beta \right)\;\delta \Delta r+\cos\kappa \;z\;\;\beta \;\delta S\\ \cos k\;x\;\delta \Delta \kappa -\sin\kappa \;\sin\left(S\beta \right)\;\delta \Delta r+\sin\kappa \;z\;\beta \;\delta S\\ -\cos\left(S\beta \right)\;\delta \Delta r-x\;\beta \;\delta S \end{array}\right] \end{array}$$

Having two conjugate points in overlapping strips, which will be denoted by subscripts *A* and *B* hereafter, the difference between the bias/noise contaminated coordinates of these points can be expressed by Equation (9). Since we are dealing with conjugate points in overlapping strips, the true coordinates of the respective points in strips *A* and *B*, *i.e.*, *X⃗*_*G_A_*_ (*True*) *and X⃗*_*G_B_*_ (*True*), should be identical. Therefore, Equation (9) would reduce to the form in Equation (10), where *e* is the combined vector of random errors. Using the established form of the impact of the biases in the system parameters on the ground coordinates (*δX⃗_G_* in Equation (8)), the discrepancy between the bias/noise contaminated coordinates of conjugate points in overlapping strips would take the form in Equation (11). In a similar fashion, if we are dealing with conjugate points in a LiDAR strip and control data, the difference between the coordinates of the control point and the bias/noise contaminated coordinates of the LiDAR point in the strip denoted by the subscript *B* can be expressed according to the form in Equation (12). Equations (11) and (12) are the final linear observation equations when dealing with overlapping strips and control data. Using conjugate points in overlapping LiDAR strips and control data, these equations allow for the recovery of biases in the system parameters (*δΔX, δΔY, δΔZ, δΔω, δΔφ, δΔκ, δΔr, δS*). Once these biases have been estimated, the adjusted coordinates of the LiDAR points can be derived according to Equation (13):

(9) $${\vec{X}}_{G_{A}}\left(b/n\right)-{\vec{X}}_{G_{B}}\left(b/n\right)={\vec{X}}_{G_{A}}\left(\mathrm{True}\right)+\delta {\vec{X}}_{G_{A}}+e_{A}-{\vec{X}}_{G_{B}}\left(\mathrm{True}\right)-\delta {\vec{X}}_{G_{B}}-e_{B}$$

(10) $${\vec{X}}_{G_{A}}\left(b/n\right)-{\vec{X}}_{G_{B}}\left(b/n\right)=\delta {\vec{X}}_{G_{A}}-\delta {\vec{X}}_{G_{B}}+e_{A}-e_{B}=\delta {\vec{X}}_{G_{A}}-\delta {\vec{X}}_{G_{B}}+e$$

(11) $$\begin{array}{c} {\vec{X}}_{G_{A}}\left(b/n\right)-{\vec{X}}_{G_{B}}\left(b/n\right)=\left[\begin{array}{c} \left(\cos\kappa_{A}-\cos\kappa_{B}\right)\delta \Delta X-\left(\sin\kappa_{A}-\sin\kappa_{B}\right)\delta \Delta Y\\ \left(\sin\kappa_{A}-\sin\kappa_{B}\right)\delta \Delta X+\left(\cos\kappa_{A}-\cos\kappa_{B}\right)\delta \Delta Y\\ 0 \end{array}\right]\\ +\left[\begin{array}{c} \left(\sin\kappa_{A}z_{A}-\sin\kappa_{B}z_{B}\right)\delta \Delta \omega +\left(\cos\kappa_{A}z_{A}-\cos\kappa_{B}z_{B}\right)\delta \Delta \varphi -\left(\sin\kappa_{A}x_{A}-\sin\kappa_{B}x_{B}\right)\delta \Delta \kappa \\ -\left(\cos\kappa_{A}z_{A}-\cos\kappa_{B}z_{B}\right)\delta \Delta \omega +\left(\sin\kappa_{A}z_{A}-\sin\kappa_{B}z_{B}\right)\delta \Delta \varphi +\left(\cos\kappa_{A}x_{A}-\cos\kappa_{B}x_{B}\right)\delta \Delta \kappa \\ -\left(x_{A}-x_{B}\right)\delta \Delta \varphi \end{array}\right]\\ +\left[\begin{array}{c} -\left[\cos k_{A}\;\sin\left(S\beta_{A}\right)-\cos\kappa_{B}\;\sin\left(S\beta_{B})\right]\delta \Delta r+\left(\cos\kappa_{A}z_{A}\beta_{A}-\cos\kappa_{B}z_{B}\beta_{B}\right)\delta S\right]\\ -\left[\sin k_{A}\;\sin\left(S\beta_{A}\right)-\sin\kappa_{B}\;\sin\left(S\beta_{B})\right]\delta \Delta r+\left(\sin\kappa_{A}z_{A}\beta_{A}-\sin\kappa_{B}z_{B}\beta_{B}\right)\delta S\right]\\ -\left[\cos\left(S\beta_{A}\right)-\cos\left(S\beta_{B})\right]\delta \Delta r-\left(x_{A}\beta_{A}-x_{B}\beta_{B}\right)\delta S\right] \end{array}\right]+e \end{array}$$

(12) $$\begin{array}{c} {\vec{X}}_{G_{\mathrm{Control}}}-{\vec{X}}_{G_{B}}\left(b/n\right)=\left[\begin{array}{c} -\cos\kappa_{B}\delta \Delta X+\sin\kappa_{B}\delta \Delta Y\\ -\sin\kappa_{B}\delta \Delta X-\cos\kappa_{B}\delta \Delta Y\\ -\delta \Delta Z \end{array}\right]\\ +\left[\begin{array}{c} -\sin\kappa_{B}z_{B}\delta \Delta \omega -\cos\kappa_{B}z_{B}\delta \Delta \varphi +\sin\kappa_{B}x_{B}\delta \Delta \kappa \\ \cos\kappa_{B}z_{B}\delta \Delta \omega -\sin\kappa_{B}z_{B}\delta \Delta \varphi -\cos\kappa_{B}x_{B}\delta \Delta \kappa \\ x_{B}\;\delta \Delta \varphi \end{array}\right]\\ +\left[\begin{array}{c} \cos\kappa_{B}\;\sin\left(S\beta_{B}\right)\delta \Delta r-\cos\kappa_{B}z_{B}\beta_{B}\;\delta S\\ \sin\kappa_{B}\;\sin\left(S\beta_{B}\right)\delta \Delta r-\sin\kappa_{B}z_{B}\beta_{B}\;\delta S\\ \cos\left(S\beta_{B}\right)\delta \Delta r+x_{B}\;\beta_{B}\;\delta S \end{array}\right]+\left(e_{\mathrm{Control}}-e_{B}\right) \end{array}$$

(13) $$\begin{array}{ll} {\left[\begin{array}{l} \tilde{X}\\ \tilde{Y}\\ \tilde{Z} \end{array}\right]}_{\mathrm{Corrected}} & ={\left[\begin{array}{l} X\\ Y\\ Z \end{array}\right]}_{\mathrm{Biased}}-\left[\begin{array}{c} \cos\kappa \;\delta \hat{\Delta }X-\sin\kappa \;\delta \hat{\Delta }Y+\sin\kappa \;z\;\delta \hat{\Delta }\omega +\cos\kappa \;z\;\delta \hat{\Delta }\varphi \\ \sin\kappa \;\delta \hat{\Delta }X+\cos\kappa \;\delta \hat{\Delta }Y-\cos\kappa \;z\;\delta \hat{\Delta }\omega +\sin\kappa \;z\;\delta \hat{\Delta }\varphi \\ \delta \hat{\Delta }Z-x\;\delta \hat{\Delta }\varphi \end{array}\right]\\ & -\left[\begin{array}{c} \;-\sin\kappa \;x\;\delta \hat{\Delta }\kappa -\cos\kappa \;\sin\left(S\beta \right)\;\delta \hat{\Delta }r+\cos\kappa \;z\;\beta \;\;\delta \hat{S}\\ \cos\kappa \;\;x\;\delta \hat{\Delta }\kappa -\sin\kappa \;\sin\left(S\beta \right)\delta \hat{\Delta }r+\sin\kappa \;z\;\beta \;\delta \hat{S}\\ -\cos\left(S\beta \right)\delta \hat{\Delta }r-x\;\;\beta \;\delta \hat{S} \end{array}\right] \end{array}$$

The procedure for estimating the quantities (*x, z, κ* and *β*) in Equations (11) and (12) using the available data (time-tagged point cloud and trajectory positions) can be summarized as follows:

- For a LiDAR point mapped at time *t*, we identify trajectory positions within a certain time interval (*t* − Δ*t*, *t* + Δ*t*);
- Then, a straight line is fitted through the selected trajectory positions to come up with a local estimate of the trajectory. After defining the local trajectory, the necessary quantities can be estimated as follows:
  ▪ *x*, which is the *x*-coordinate of the LiDAR point with respect to the laser unit coordinate system, *i.e.*, the lateral distance with the appropriate sign between the LiDAR point in question and the projection of the flight trajectory onto the ground, can be determined by computing the normal distance between the LiDAR point and the interpolated trajectory that have been projected onto the ground;
  ▪ *z*, which is the vertical coordinate of the LiDAR point with respect to the laser unit coordinate system, can be determine by subtracting the elevation of the laser beam firing point at time *t*, given by the interpolated flight trajectory, from the LiDAR point elevation;
  ▪ *κ,* which is the trajectory heading, can be computed once we have the local estimate of the trajectory and its direction (defined by the neighboring trajectory positions); and
  ▪ *β*, which is the scan angle, can be computed by simple trigonometric operation using the estimated lateral distance *x* and the trajectory height above ground *z.*

### 2.2. Remarks Regarding the Necessary Flight and Control Configuration for LiDAR Calibration

Analyzing the mathematical expressions for the discrepancies between bias/noise contaminated coordinates of conjugate points in overlapping strips—Equation (11)—as well as the discrepancies when dealing with control data—Equation (12), one can make the following observations/recommendations regarding the preferred flight and ground control configuration for reliable estimation of biases in the system parameters:

1. Using only overlapping strips, one cannot estimate the vertical bias in the lever arm *δΔZ*—this term is absent in Equation (11). Such inability is caused by the fact that a vertical bias in the lever arm produces the same effect regardless of the flying direction, flying height, or scan angle—refer to Equation (8). In other words, a bias in the vertical component of the lever arm would not lead to discrepancies among overlapping strips regardless of the flight configuration.
2. The impact of the range bias *δΔr* is flight direction independent for parallel flight lines (*i.e.*, the impact is identical for flight lines flown in opposite directions). As can be seen in Equation (8), the range bias affects the coordinates of the points in the vertical direction (major component) as well as the planimetric component across the flight direction (minor component)—sin(*Sβ*) ≪ cos(*Sβ*) in the range of the utilized scan angles (e.g., *β* ∈ [−25°, + 25°]). Therefore, the vertical discrepancy is expected to be more useful than the planimetric discrepancy for the estimation of the range bias. However, the vertical discrepancy – which is caused by the range bias—among overlapping strips will be almost zero since cos(*Sβ*)≈ 1 for the above mentioned scan angle range. Therefore, the estimation of the range bias would be quite difficult to estimate by evaluating/observing the discrepancies among overlapping strips.
3. The impacts of biases in the boresight heading angle *δΔκ* and the mirror angle scale *δS* are flight direction independent for parallel flight lines (*i.e.*, the impact is identical for flight lines flown in opposite directions).
4. Having opposite flight lines with 100% overlap is optimal for the recovery of the planimetric biases in the lever arm (*δ*Δ*X*, *δ*Δ*Y*) as well as the biases in the boresight pitch and roll angles (*δ*Δ*ω*, *δ*Δ*φ*). In such a case, the impact of these biases on the strip-pairs will be doubled while the discrepancies arising from other biases will be eliminated.
5. The impact of biases in the lever arm on the introduced discrepancies is independent of the flying height. However, the impact of the biases in the boresight angles increases with an increase in the flying height. Moreover, for a given flight height and relatively flat terrain (*i.e.*, *z_A_ and z_B_* are almost constant in the overlap area), there is a strong correlation between the biases in boresight pitch *δ*Δ*ω* and the planimetric component of the lever arm in the flight direction *δ*Δ*Y*. To avoid such a correlation, it is recommended to have opposite flight lines with 100% overlap ratio captured at different flying heights. Larger height difference between the flight pairs would be recommended as long as the point density in the higher flight lines is not too low to jeopardize the ability of identifying conjugate primitives.
6. Having two parallel flight lines with less than 100% overlap is necessary for the estimation of the bias in the boresight heading angle *δ*Δ*κ*. For such a case, biases in the lever arm would not lead to any discrepancies among overlapping strips. Moreover, biases in the boresight pitch and roll angles (*δ*Δ*ω* and *δ*Δ*φ*, respectively) would not lead to any planimetric discrepancies among conjugate points in overlapping strips. The bias in the boresight heading angle *δ*Δ*κ* would cause a constant planimetric discrepancy among overlapping strips (along the flight direction). The bias in the boresight roll angle *δ*Δ*φ* would cause a constant vertical discrepancy among overlapping strips. An increase in the lateral distance between the parallel strips would lead to larger discrepancies, which in turn will have a positive impact on the reliability of the boresight heading and roll bias estimation (*for parallel flight lines in the same direction*, *x_A_* − *x_B_* *= lateral distance between the strips*).
7. To estimate biases in the vertical component of the lever arm *δ*Δ*Z* and range measurement *δ*Δ*r*, control information is necessary. For these biases, vertical control would be sufficient since it will allow us to observe the discrepancy between the LiDAR and the control surfaces in the vertical direction—refer to Equation (12). Having said that, one should note that there will be high correlation between the impacts of these biases. As can be observed in the third line in Equation (12), the impact of the range bias (*cos*(*Sβ*) *δΔr*) is almost constant in the range of the utilized scan angles (*i.e.*, *cos*(*Sβ*) ≈ 1 for *β* ∈ [−25°, + 25°) and therefore, it will be correlated with the impact of the bias in the vertical component of the lever arm (−*δ*Δ*Z*). To avoid such a problem, one can rely on *in-situ* measurements of the vertical lever arm and only solving for the range bias during the calibration process.
8. For reliable estimation of the bias in the mirror angle scale *δS*, well distributed data in the across flight direction should be used (this distribution will exhibit the nonlinear discrepancy pattern associated with this bias as can be seen in Equations (11) and (12)).

Based on the above discussion, one can conclude that the optimal/minimal flight and control configuration for the estimation of the system parameters consists of three overlapping pairs and one vertical control point. More specifically, two strip pairs, which are captured from two flying heights in opposite directions with 100% overlap, and two parallel flight lines, which are flown in the same direction with the least overlap possible, are needed. As for the parameters to be estimated, due to high correlation, it is not recommended to simultaneously solve for the vertical bias in the lever arm *δΔZ* and the range error *δ*Δ*r.*

### 2.3. Least Squares Adjustment Model and Primitives

The mathematical model that has been developed so far is based on the availability of conjugate points in overlapping strips, Equation (11), or a LiDAR strip and a control surface, Equation (12). Assuming that such conjugate points exist, observations representing the discrepancy between these points follow the traditional Gauss Markov Stochastic model in Equation (14). The Least Squares Adjustment (LSA) procedure aims at estimating the unknown parameters, which minimize the sum of squares of weighted residuals in Equation (15), which would lead to the solution in Equations (16–19) [25]:

(14) $$:y=\mathrm{Ax}+e\;\;\;\;\;\;\;\;\;\;e∼\left(0,\Sigma \right)\;\;\;\;\;\;\;\;\;\;\mathrm{where}\;\;\;\;\;\;\;\;\;\Sigma =\sigma_{o}^{2}P^{-1}$$

where

– *y* is the *nx1* vector of observations (discrepancies between conjugate points),
– *x* is the *mx1* vector of unknowns (biases in the system parameters),
– *A* is the *nxm* design matrix, and
– *e* is the *nx1* vector of random noise, which is normally distributed with a zero mean and variance-covariance matrix Σ
– $\sigma_{o}^{2}$ is the a-priori variance factor
– *P* is the weight matrix of the noise vector

(15) $$e^{T}\mathrm{Pe}=\min{|}_{x}\;\;\;\;\;\;\;\;\;\;\;\;\;\;\;\;\;\;\;\;\;\;\;\left(\text{LSA Target Function}\right)$$

(16) $$\hat{x}={\left(A^{T}\mathrm{PA}\right)}^{-1}A^{T}\mathrm{Py}=N^{-1}A^{T}\mathrm{Py}\;\;\;\;\;\;\;\;\;\;\;\;\;\;\;\;\;\left(\text{Solution Vector}\right)$$

(17) $$\tilde{e}=y-A\hat{x}\;\;\;\;\;\;\;\;\;\;\;\;\;\;\;\;\;\;\;\;\;\;\;\;\;\;\left(\text{Predicted Residuals}\right)$$

(18) $$\sum \left\{\hat{x}\right\}={\hat{\sigma }}_{o}^{2}{\left(A^{T}\mathrm{PA}\right)}^{-1}={\hat{\sigma }}_{o}^{2}N^{-1}\;\;\;\;\;\;\;\;\;\;\;\;\;\;\;\;\;\;\;\;\;\;\;\;\left(\text{Variance-Covariance Matrix}\right)$$

(19) $${\hat{\sigma }}_{o}^{2}=\left({\tilde{e}}^{T}P\tilde{e}\right)/\left(n-m\right)\;\;\;\;\;\;\;\;\;\;\;\;\;\;\;\;\;\;\;\;\;\;\;\;\;\;\;\left(\text{A-posteriori Variance Factor}\right)$$

For LiDAR data, there is no point-to-point correspondence between overlapping strips or between a given strip and a control surface. Therefore, the abovementioned LSA solution cannot be used to come up with an estimate of the biases in the system parameters. Therefore, the following subsections will deal with the necessary modification to the stochastic model in Equation (14) to allow for the estimation of biases in the system parameters without having conjugate points in overlapping strips. First, we will discuss the conjugate primitives that could be identified in overlapping LiDAR strips (point-patch pairs). Then, we will discuss the modification of the stochastic model that would allow for the utilization of these conjugate primitives for the estimation of biases in the system parameters.

#### 2.3.1. Proposed Primitives

**Figure 2.**
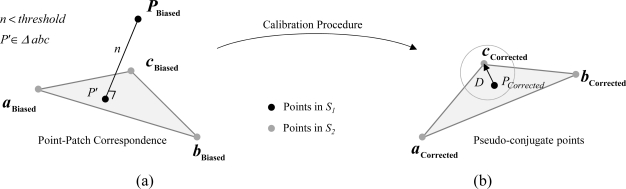
(**a**) Point-patch correspondence procedure and (**b**) the additional unknown vector *D* following the calibration procedure.

In this research, one of the strips, denoted by *S_1_*, is represented by the original points while the second strip, denoted by *S_2_*, is represented by triangular patches, which can be derived from a TIN generation procedure. When control surface is used, it will be represented by the original control points (due to its sparse nature) and the LiDAR strips will be represented by triangular patches. It is important to note that the utilized discrete control points do not need be identifiable in the LiDAR surfaces. Having TIN patches with varying orientation (slope and aspect) overlapping the control points will contribute towards better estimation of all biases. The correspondence between points in *S_1_* and patches in *S_2_* is established using the closest patch procedure as shown in [26–27]. In this procedure, a TIN patch is deemed conjugate to a given point if it is the closest patch to this point. In addition, the point-patch separation (normal distance *n*) should not exceed a pre-defined threshold. Finally, the point projection onto the patch should be located inside the TIN patch (Figure 2a).

For a given point-patch pair, we will assume that one of the vertices of the TIN patch in *S_2_* is conjugate to the corresponding point in *S_1_*. Note that any vertex of the TIN patch can be selected, as will be clarified in the next section. The TIN vertex in *S_2_* and the point in *S_1_* for a point-patch pair are denoted as pseudo-conjugate points (Figure 2b). Starting from Equation (9), the mathematical model describing the discrepancy between pseudo-conjugate points will take the form in Equation (20). The stochastic model describing the discrepancy in Equation (20) can be represented by the Gauss Markov Stochastic model in Equation (21). The difference between this model and the one in Equation (14) is the additional unknown vector *D* resulting from using non-conjugate points along a point-patch pair (Figure 2b). It should be noted that the additional unknown vector *D* is in the plane of the TIN patch under consideration (*i.e.*, the component of this vector along the normal to the TIN patch is zero):

(20) $$\begin{array}{l} {\vec{X}}_{G_{A}}\left(b/n\right)-{\vec{X}}_{G_{B}}\left(b/n\right)={\vec{X}}_{G_{A}}\left(\mathrm{True}\right)+\delta {\vec{X}}_{G_{A}}-{\vec{X}}_{G_{B}}\left(\mathrm{True}\right)-\delta {\vec{X}}_{GB}+e\\ \;\;\;\;\;\;\;\;\;\;\;\;\;\;\;\;\;\;\;\;\;\;\;\;\;\;\;\;\;\;\;\;\;\;=\delta {\vec{X}}_{G_{A}}-\delta {\vec{X}}_{G_{B}}+D+e \end{array}$$

where

(21) $$\begin{array}{l} -D={\vec{X}}_{G_{A}}\left(\mathrm{True}\right)-{\vec{X}}_{G_{B}}\left(\mathrm{True}\right)\\ \;\;\;\;\;\;\;\;\;\;\;\;\;\;\;\;\;\;\;\;\;\;\;\;\;\;\;\;\;\;\;\;\;\;\;\;\;\;\;\;\;\;y=\mathrm{Ax}+D+e\;\;\;\;e∼\;\left(0,\sum \right)\;\;\;\;\mathrm{where}\;\sum =\sigma_{o}^{2}P^{-1} \end{array}$$

#### 2.3.2. Modified Least Squares Adjustment

The main objective for the development of the modified LSA is to deal with the model in Equation (21) while eliminating the unknown vector *D* from the parameters to be estimated. To explain the modification process, we will start by changing the stochastic properties of the random noise vector as represented by Equation (22). As it can be seen in this equation, the unknown vector *D* belongs to the null space of the modified weight matrix *P*^′^. Such a condition signifies that the modified weight matrix is not positive-definite (*i.e.*, the inverse matrix *P*^′−1^ does not exist). Therefore, the modified variance-covariance matrix will be represented as follows: $\Sigma^{\prime }\{e\}=\sigma_{o}^{2}P^{\prime +}$, where the plus sign indicates the Moore-Penrose pseudoinverse). Starting from the modified variance-covariance/weight matrix and the LSA principles, the solution will be derived below:

(22) $$\sum^{\prime }\left\{e\right\}=\sigma_{o}^{2}P^{\prime +}\;\;\;\;\text{where}\;\;\;\;\;P^{\prime }D=0$$

Using the modified weight matrix, the LSA target function can be redefined as *per* Equation (23). Since the additional unknown vector *D* belongs to the null space of the modified weight matrix (*i.e.*, *P*^′^*D* = 0), then the LSA target function in Equation (23) reduces to the form in Equation (24). Thus, the solution *x̂* to the LSA target function is defined by Equation (25), refer to Appendix A for more details. Using the law of error propagation, the variance-covariance matrix of the solution vector Σ{*x̂*} is shown in Equation (26), refer to Appendix A for the detailed derivation:

(23) $$e^{T}P^{\prime }e={\left(y-\mathrm{Ax}-D\right)}^{T}P^{\prime }\left(y-\mathrm{Ax}-D\right)=\min{|}_{x,D}\;\;\;\;\;\;\;\;\text{LSA Target Function}$$

(24) $$e^{T}P^{\prime }e={\left(y-\mathrm{Ax}\right)}^{T}P^{\prime }\left(y-\mathrm{Ax}\right)=\min{|}_{x}\;\;\;\;\;\;\;\;\text{LSA Target Function}$$

(25) $$\hat{x}={\left(A^{T}P^{\prime }A\right)}^{-1}A^{T}P^{\prime }y=N^{-1}A^{T}P^{\prime }y\;\;\;\;\;\;\;\;\;\;\;\;\;\;\;\;\;\;\;\text{Estimated Unknowns}$$

(26) $$\sum \left\{\hat{x}\right\}=\sigma_{o}^{2}N^{-1}\;\;\;\;\;\;\;\;\;\;\;\;\;\;\;\;\;\;\;\;\;\;\;\;\;\;\;\;\;\;\;\;\;\text{Varinance-Covariance Matrix}$$

The last step is to estimate the a-posteriori variance factor ${\hat{\sigma }}_{o}^{2}$ by deriving the expected value of the sum of squares of weighted predicted residuals. Starting from Equation (27), one can derive an estimate for the a-posteriori variance factor according to Equation (28), where *q* is the rank of the modified weight matrix *P*^′^ – refer to Appendix A for the detailed derivation:

(27) $$E\left({\tilde{e}}^{T}P^{\prime }\tilde{e}\right)=E\left\{{\left(y-A\hat{x}-D\right)}^{T}P^{\prime }\left(y-A\hat{x}-D\right)\right\}=E\left\{{\left(y-A\hat{x}\right)}^{T}P^{\prime }\left(y-A\hat{x}\right)\right\}=\left(q-m\right)\sigma_{o}^{2}$$

(28) $${\hat{\sigma }}_{o}^{2}={\left(y-A\hat{x}\right)}^{T}P^{\prime }\left(y-A\hat{x}\right)/\left(q-m\right)$$

In summary, from an implementation point of view, the LSA solution to the stochastic model in Equation (29) can be derived using Equations (25), (26) and (28). This solution is similar to that of the traditional Gauss Markov model (Equations (16), (18) and (19)) with the exception that the redundancy is evaluated as the difference between the rank of the modified weight matrix and the number of unknowns. Thus, the modification in the weights of the noise vector allows for the elimination of the additional unknown vector *D* while having almost no impact on the traditional LSA (*i.e.*, the solution is obtained using the traditional solution for the Gauss Markov Model in the absence of the additional unknown vector):

(29) $$y=\mathrm{Ax}+D+e\;\;\;\;e∼\left(0,\sum^{\prime }\right)\;\;\;\mathrm{where}\;\sum^{\prime }=\sigma_{o}^{2}P^{\prime +}\mathrm{and}\;P^{\prime }D=0$$

So far, we established that by modifying the weight matrix to satisfy the condition in Equation (22), one can derive an estimate of the biases in the system parameters while dealing with non-conjugate points along corresponding point-patch pairs. The question now is how to derive the modified weight matrix *P*^′^. This can be established according the following procedure. First, one starts by defining a new coordinate system (*UVW*), where the *UV* axes are aligned along the TIN patch involved in the current point-patch pair (*i.e.*, W is parallel to the TIN patch normal). The relationship between the *XYZ* and *UVW* coordinates—assuming that the two systems share the same origin—can be expressed by Equation (30). The rotation matrix *M* can be defined using the direction of the normal to the TIN patch as well as the direction of the *U* and *V* axes along the TIN plane (one should note that the choice of the *U* or *V* directions is quite arbitrary):

(30) $$\left[\begin{array}{c} U\\ V\\ W \end{array}\right]=M\left[\begin{array}{c} X\\ Y\\ Z \end{array}\right]$$

The weight matrix of the transformed coordinates in the *UVW* system can be derived from the law of error propagation according to Equation (31). This weight matrix is then modified according to Equation (32):

(31) $$P_{\mathrm{UVW}}=MP_{\mathrm{XYZ}}M^{T}=\left[\begin{array}{ccc} P_{U} & P_{\mathrm{UV}} & P_{\mathrm{UW}}\\ P_{\mathrm{VU}} & P_{V} & P_{\mathrm{VW}}\\ P_{\mathrm{WU}} & P_{\mathrm{WV}} & P_{W} \end{array}\right]$$

(32) $$P_{\mathrm{UVW}}^{\prime }=\left[\begin{array}{ccc} 0 & 0 & 0\\ 0 & 0 & 0\\ 0 & 0 & P_{W} \end{array}\right]$$

Finally, the modified weight matrix in the *XYZ* coordinate system is defined by Equation (33). Using the modified weight matrix in Equation (33), one can show that *P*^′^*_XYZ_D* =0—Refer to Equations (34) while noting that the *D* vector is aligned along the TIN plane (refer to Figure 2b). It should be noted that the weight modification will be carried out for all the TIN patches of the established correspondences; whether for point-patch pairs in overlapping strips or point-patch pairs in LiDAR and control surfaces:

(33) $$P_{\mathrm{XYZ}}^{\prime }=M^{T}P_{\mathrm{UVM}}^{\prime }M$$

(34) $$P_{\mathrm{XYZ}}^{\prime }D=M^{T}P_{\mathrm{UVM}}^{\prime }M\left[\begin{array}{c} \mathrm{dX}\\ \mathrm{dY}\\ \mathrm{dZ} \end{array}\right]=M^{T}P_{\mathrm{UVM}}^{\prime }\left[\begin{array}{c} \mathrm{dU}\\ \mathrm{dV}\\ 0 \end{array}\right]=M^{T}\left[\begin{array}{ccc} 0 & 0 & 0\\ 0 & 0 & 0\\ 0 & 0 & P_{W} \end{array}\right]\;\left[\begin{array}{c} \mathrm{dU}\\ \mathrm{dV}\\ 0 \end{array}\right]=0$$

In summary, the proposed Quasi-rigorous calibration procedure proceeds as follows:

1. The correspondence between points in *S_1_* and patches in *S_2_* is established using the closest patch procedure as explained in [26] and [27].
2. For each conjugate point-patch pair from overlapping LiDAR strips, (e.g., patch vertex *X⃗*_*G*_*B*__(*b/n*) in *S_2_* and point *X⃗*_*G*_*A*__(*b/n*) in *S_1_*), one can write the observation equations similar to those in Equation (11).
3. When a control surface is used, it is represented by the original points (due to its sparse nature) and the LiDAR strips are represented by triangular patches. Then, for each conjugate point-patch pair, one can write the observation equations similar to those in Equation (12). It is important to mention that the observation equations can be written for all LiDAR strips which overlap with the control surface.
4. For each conjugate point-patch pair, the weight of the observed discrepancy vector *X⃗*_*G*_*A*__(*b/n*) − *X⃗*_*G*_*B*__(*b/n*) should be modified using Equations (31–33).
5. The established correspondences and the corresponding observations with their modified weight matrices are used to derive an estimate of the system biases. Then, the estimated system biases are used to reconstruct an adjusted point cloud using Equation (13).
6. Following the reconstruction of the LiDAR point cloud, the correspondence between point-patch pairs might change. Therefore, a new set of correspondences has to be established. The new correspondences are then utilized to derive a better estimate of the system biases.
7. Such a procedure is repeated until the corrections to the estimated calibration parameters are almost zero.

One should note that each pair of pseudo-conjugate points provides three observations of the form in Equation (11) or Equation (12). However, these three observations increase the redundancy by only one (the rank of the modified weight matrix for these three equations is one—refer to Equation (32)). In other words, the three observations would increase the redundancy by three if and only if the 3D discrepancy between the pseudo-conjugate points is considered. After the weight modification process, the 2D discrepancy between the pseudo-conjugate points along the TIN plane (the unknown vector *D* is ignored during the minimization process. Therefore, only the discrepancy between the pseudo-conjugate points along the TIN normal is only minimized during the modified LSA (thus, the three observations only increase the redundancy by one). Since the modified LSA only minimizes the normal distance between a point in *S_1_* and the corresponding TIN in *S_2_*, the topography in the overlap area should have different slope and aspect values to ensure reliable estimates of the bias parameters.

### 2.4. Impact of the Geometric Calibration on the Relative and Absolute Accuracy

The impact of the geometric calibration on the relative accuracy of the LiDAR point cloud can be assessed by checking the degree of compatibility between conjugate surface elements in overlapping strips before and after reconstructing the point cloud using the estimated biases. In this work, the compatibility will be evaluated qualitatively and quantitatively. The qualitative evaluation will be performed by visual inspection of profiles generated using the original and adjusted point cloud to check any improvements in the quality of fit between overlapping strips. The quantitative assessment, on the other hand, will be performed by computing the necessary 3D transformation parameters for the co-alignment of overlapping strips before and after the calibration procedure. For the computation of the 3D transformation parameters, the proposed Iterative Closest Patch (ICPatch) procedure in [26] and [27] will be employed.

The impact of the geometric calibration on the absolute accuracy can be evaluated by quantifying the degree of compatibility between LiDAR and control surfaces before and after the calibration process. From an implementation point of view, such a procedure would not always be feasible due to the control surface requirement. In this work, linear features extracted from the LiDAR data before and after the calibration process are used for the geo-referencing of an image block covering the same area. The absolute accuracy of the derived ground coordinates from the geo-referenced image block is evaluated using a check point analysis. The adopted methodologies for linear features extraction and their utilization for the photogrammetric geo-referencing are detailed in [27] and [28], respectively.

## 3. Radiometric Correction and Land Cover Classification

The physical properties of the laser energy are considered with respect to the sensor configuration and environmental parameters using the radar equation, which is proposed to model the power of the received signal [29]. The radar equation can be presented as follows:

(35) $$P_{r}=\frac{P_{t}D_{r}^{2}}{4\pi r^{4}\beta_{t}^{2}}\eta_{\mathrm{sys}}\eta_{\mathrm{atm}}\sigma$$

In this equation, the received signal power *P_r_* depends on the transmitted signal power *P_t_*, receiver aperture diameter *D_r_*, range from the sensor to the target *r*, laser beam width *β_t_*, system-specific factor *η_sys_*, atmospheric transmission factor *η_atm_*, and target (backscattering) cross-section σ, which depends on the target characteristics and is defined as follows:

(36) $$\sigma =\frac{4\pi }{\Omega }\rho_{s}A_{s}$$

In the above equation, Ω is the scattering solid angle, *A_s_* is the target area, and *ρ_s_* is the target spectral reflectance.

The radiometric correction aims at converting the intensity value *I* into the spectral reflectance *ρ_s_* by considering the parameters described in the radar equation. It is assumed that the intensity *I* represents the peak value of *P_r_* and the LiDAR data providers linearly transform *P_r_* into 8 bit values to represent the intensity data, which is used in the radar equation [17]. The receiver aperture diameter *D_r_*, laser beam width *β_t_*, and system-specific factor *η_sys_* are considered to be constant during the flight mission. The range *r* of each laser pulse is the distance between the instantaneous location of the sensor and the target. This range is critical for the radiometric correction since the attenuation of the laser energy is directly proportional to the distance of travel [19]. The scattering solid angle is derived as the cosine of the angle between the surface normal and the direction of the laser pulse [30] based on the assumption of a Lambertian reflectance. The transmitted laser energy *P_t_*, though is usually unknown, can be assumed as constant or can be related to the pulse repetition frequency (PRF) [20]. The atmospheric attenuation *η_atm_* depends on the temperature, pressure, and humidity during the flight survey. These parameters can be obtained from the nearest weather observatory station. Details on the radiometric correction process can be found in [31].

Land cover classification is conducted to evaluate the impact of the geometric calibration and the radiometric correction of the LiDAR data on the final data products. Two datasets are prepared from the original and modified LiDAR data after geometric calibration and radiometric correction. Each set of data includes the interpolated digital surface model and the interpolated intensity. Four land cover classes (tree, grass, soil and built-up area) are identified in both datasets. Using the same training sites, Maximum likelihood classification is conducted on both datasets and finally the classification results are evaluated by conducting an accuracy assessment using check points, which can be generated from an orthorectified aerial photo. The overall accuracy and kappa coefficient are used to evaluate the classification results.

## 4. Study Area and Dataset

**Figure 3.**
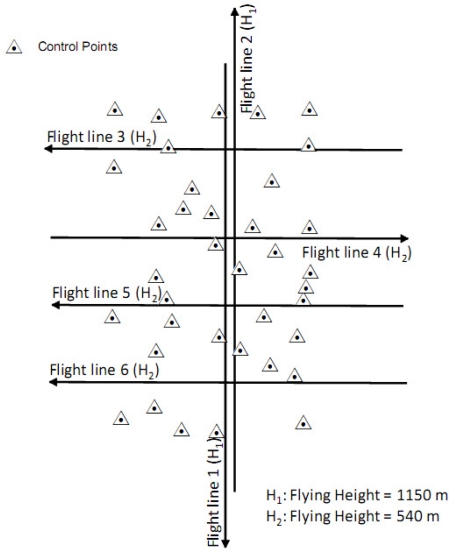
Flight and control configuration of the LiDAR dataset.

A real LiDAR dataset was acquired to test the feasibility of the proposed methods. The study area covers the British Columbia Institute of Technology (BCIT) located at Burnaby, British Columbia, Canada (122°59′W, 49°15′N). The area contains buildings and parking lots connected by sidewalks and paved road segments. Individual shrubs and open spaces covered by grass can also be found in the surveyed area. The LiDAR mission was conducted on July 17, 2009 from 14:37 to 15:15 local time. The day of the mission was a sunny day with a temperature of 29.8 °C. The visibility and the pressure were 48.3 km and 101.81 kPa, respectively, as delivered by the National Climate Data and Information Archive from Environment Canada. The LiDAR sensor used was a Leica ALS50 operating at a 1.064 μm wavelength with 0.33 mrad beam divergence. The captured LiDAR data consists of six strips that cover a 1 km by 2 km area. The configuration of the flight lines is shown in Figure 3. The acquired data consists of a 3D point cloud with multiple returns in LAS format together with the trajectory data. The average point density of the LiDAR data acquired using the flying height H_1_ (1150 m) is 1.5 points/m^2^ while for the flying height H_2_ (540 m) the average point density is 3.7 points/m^2^. In the surveyed area, thirty-seven control points were established by a GPS survey. These control points were used for the geometric calibration and check point analysis to evaluate the absolute accuracy of the adjusted point cloud.

## 5. Results and Discussion

### 5.1. Geometric Calibration

In addition to testing the feasibility of the proposed geometric calibration procedure, we would like to investigate whether the calibration results are significantly different when using more overlapping strip-pairs than the minimum recommended configuration as discussed in Section 2.2. Table 1 shows the different investigated scenarios. Experiment “I” utilizes all the available overlapping strip-pairs (4 overlapping strip-pairs) without any control information. Experiment “II”, on the other hand, utilizes all the available overlapping strip-pairs and 37 vertical ground control points. Experiment “III” utilizes the 4 overlapping strip-pairs with only 1 vertical control point. Finally, experiment “IV” utilizes the minimum recommended overlapping strip-pairs configuration (3 overlapping strip-pairs) and 37 vertical ground control points.

As already mentioned, for the estimation of the biases in the vertical component of the lever arm (*δ*Δ*Z*) and range measurement (*δ*Δ*r*), control information is necessary. However, they cannot be estimated simultaneously due to the high correlation among them. Therefore, in the calibration process, we only solve for the range bias while relying on the field/*in-situ* measurements for the vertical component of the lever arm. For reliable estimation of the bias in the mirror angle scale (*δS*), well distributed data in the overlapping area in the across flight direction was manually selected for the calibration process. Table 2 reports the estimated biases in the system parameters for the different experiments. One can note in Table 2 that a significant bias in the boresight roll angle (*δ*Δ*φ*) is detected in all experiments (refer to the highlighted cells in Table 2). One can also observe a non-negligible bias in the boresight pitch angle (*δ*Δ*ω*). By comparing experiments “I” and “II”, we can observe compatible results for the estimated mounting parameters. This is expected, since the addition of vertical control information will mainly contribute to the estimation of the range bias. When comparing experiments “II” and “III”, we can note that with the reduction in the number of utilized GCP, the estimated range bias is not significantly changed. Hence, one can conclude that a single vertical GCP would be sufficient for the estimation of the range bias as long as it is observed in several strips (in this dataset, the control point was visible in 5 strips). Finally, experiment IV shows that the recommended minimum flight configuration is capable of producing reliable estimates for the biases in question. In experiment IV, the estimated parameters, which are showing the largest variation when compared to the estimated parameters in experiments I, II, and III, are the boresight pitch and heading biases. This can be attributed to a correlation of 0.63 between the boresight heading and pitch biases and a correlation of 0.72 between the boresight heading bias and the planimetric component of the lever arm in the along-flight direction. In experiments I, II, and III, these correlations are close to zero. This increased correlation can be explained by the fact that the overlapping strip-pair 5&6, which is the overlapping strip-pair whose configuration contributes most towards the estimation of the boresight heading bias (*i.e.*, parallel strips flown in the same direction), didn’t have enough features in the overlapping region with topography/buildings exhibiting varying slope and aspect. Therefore the addition of one more overlapping strip-pair improved the results.

### 5.2. Impact of the Geometric Calibration on the Relative and Absolute Accuracy

**Figure 4.**
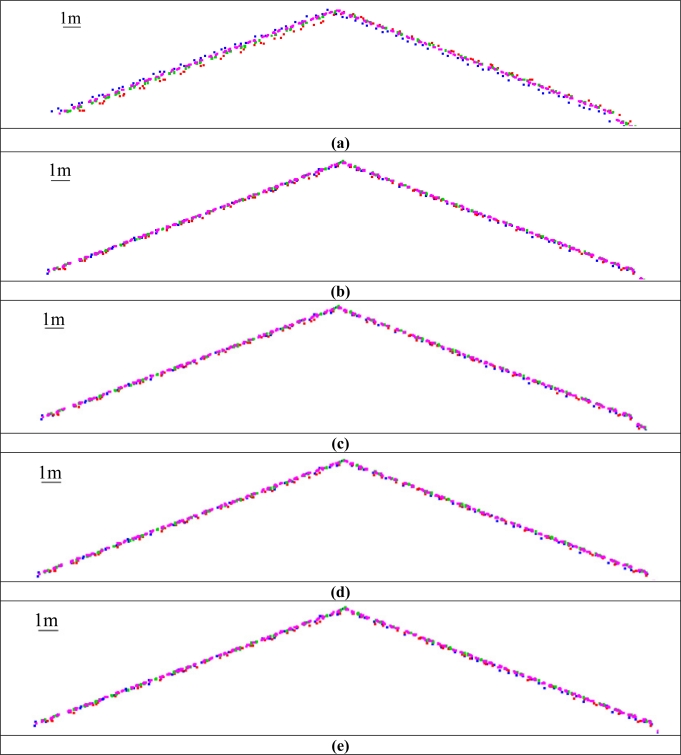
Profiles along the *X* direction over a building covered by strips “1” (in blue), “2” (in red), “3” (in green), and “4” (in magenta); (**a**) Before the calibration procedure; (**b**) After the calibration procedure using the configuration in “I”; (**c**) After the calibration procedure using the configuration in “II”; (**d**) After the calibration procedure using the configuration in “III”; and (**e**) After the calibration procedure using the configuration in “IV”.

To evaluate the impact of the geometric calibration on the relative accuracy, the compatibility of overlapping strips before and after the calibration procedure (using the different experiment scenarios) is assessed. The compatibility of the point cloud is evaluated qualitatively and quantitatively. The qualitative evaluation is performed by visual inspection of profiles generated using the original and adjusted point cloud to check any improvements in the quality of fit between overlapping strips. The improvement in the strips compatibility is illustrated in Figure 4, which shows a profile covered by strips 1 (in blue), 2 (in red), 3 (in green), and 4 (in magenta) along the *X* direction before and after the calibration procedure using the different scenarios. The quantitative assessment, on the other hand, is performed by computing the necessary 3D transformation parameters for the co-alignment of overlapping strips before and after the calibration procedure. The computed transformation parameters are reported in Table 3. As it can be seen in this table, large discrepancies can be observed before the calibration procedure, especially in the across flight direction between strips flown in opposite directions (*X_T_* direction for strips 1&2, and *Y_T_* direction for strips 3&4 and strips 4&5—refer to the circled cells in Table 3). This is expected since a larger bias was estimated in the boresight roll angle, which mainly affects the across-flight direction, *i.e.*, constant shift across the flight direction and a rotation around the flight direction [27]. One can also observe significant improvement after the calibration procedure for such strips in the across and along flight directions (refer to the highlighted cells in Table 3) due to the accurate estimation of the biases in the boresight roll and pitch angles. On the other hand, minor improvement can be observed for strips 5&6. This is due to the fact that for strips flown in the same direction the boresight roll bias only causes a constant vertical shift between conjugate surfaces elements with a much smaller magnitude while the boresight pitch bias would not lead to any discrepancies.

The qualitative and quantitative evaluations demonstrate compatible results from the different investigated scenarios. One should finally note that the range bias, which was removed in the experiments using control information, does not lead to significant discrepancies among conjugate surface elements in overlapping strips. Therefore, we cannot evaluate the introduced improvement when adding the range bias in the calibration process by checking the compatibility among overlapping strip-pairs. Such an analysis is done next in the absolute accuracy verification.

For all investigated scenarios, significant improvement in the planimetric accuracy can be observed (refer to the highlighted cells). This can be explained by the fact that the main detected bias in the studied dataset is in the boresight roll and pitch angles, which mostly affect the horizontal accuracy. One can also note that when reducing the number of utilized overlapping strip-pairs (experiment IV), the results are not negatively affected. For the experiments using control information (experiments II, III, and IV), where we solved for the range bias, we can observe that the bias value in the vertical direction has been significantly reduced (refer to the circled values in Table 4). Similar to what has been established in the relative accuracy evaluation, increasing the number of ground control points did not improve the absolute accuracy.

### 5.3. Radiometric Correction

**Figure 5.**
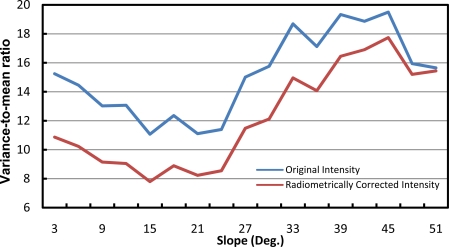
Variance-to-mean ratio of the intensity data (before and after radiometric correction) for different slopes.

To assess the impact of radiometric correction, the variance-to-mean ratio of the intensity values for the whole dataset are plotted against the surface slope before and after the radiometric correction (Figure 5). The variance-to-mean ratio represents the inverse of the signal-to-noise ratio where small amount of variance-to-mean ratio implies less noise within the data. Therefore, a reduction in the variance-to-mean ratio indicates a reduction in the noise within the intensity values. The purpose of plotting this ratio against the slope is to evaluate the variation of the intensity values with respect to the different topographic conditions. As can be seen in Figure 5, the variance-to-mean ratio is reduced after the radiometric correction by approximately 20% to 30% (therefore, improved signal content is achieved by the radiometric correction). In general, the reduction is larger when dealing with slopes of 30° or less. For slopes greater than 30°, less pronounced reduction can be observed. This can be explained by the fact that the majority of features with slope greater than 30° belong to tree clusters where high variance of the intensity value is expected due to the irregular tree canopy.

To further assess the impact of the radiometric correction, the intensity values before and after radiometric correction are compared for different land cover classes that have been identified with the help of an overlapping orthophoto of the study area. This comparison is conducted to check whether the proposed procedure would have any impact on the homogeneity and separability of the investigated classes. Table 5 shows the mean and standard deviation of the intensity values for four land cover classes, which are used to investigate if there is any overlap of intensity values amongst different land cover features. In the original intensity data, there is an overlap amongst all the land cover classes. After radiometric correction, the mean values of the built-up areas, grassland, and soil are reduced by 17% to 24%. The standard deviation values are also reduced for these three classes by 20% to 47%. There is also significant change in the intensity and standard deviation values for the grassland and tree classes. For the tree class, the standard deviation after radiometric correction is significantly increased (from 9.3 to 44.2) and the mean intensity value changed from 21.4 to 82.7. Despite this significant increase in the standard deviation of the intensity value after radiometric correction, the intensity range of the tree class does not have any overlap with the other three land cover classes. These results indicate that the homogeneity within most of the specified land cover classes is enhanced and the separability amongst all the different land cover classes is improved.

### 5.4. Impact of the Geometric Calibration and Radiometric Correction on Land Cover Classification

Using more than 1,000 check points that have been identified with the help of an orthophoto over the study area, we compared the four-land cover classification results (Tree, Built-up Area, Grass, and Soil) with the manual classification of the check points. Tables 6 and 7 show the confusion matrix, overall accuracy, and kappa coefficient of the classification results for the original and the geometrically calibrated/radiometrically corrected LiDAR data.

The overall classification accuracy using the intensity from the original LiDAR data is about 63.0%. After geometric calibration and radiometric correction, the overall accuracy increased to 70.5%. The kappa coefficient has also increased from 0.442 to 0.558. Considering each individual land cover class, the kappa coefficient of the tree and built-up features are found to be always lower than 0.5 using the original LiDAR dataset. After geometric calibration and radiometric correction, the kappa coefficient of the tree class increases from 0.464 to 0.725 and the kappa coefficient of the built-up class increases from 0.486 to 0.593. This improvement in the classification results is due to the high separability of the intensity values between different class features after the radiometric correction. A slight improvement of the kappa coefficient of the grass class can also be detected after the geometric calibration and radiometric correction. It can be also observed that the proposed process helps in reducing the confusion between grass and tree classes in LiDAR data classification.

**Figure 6.**
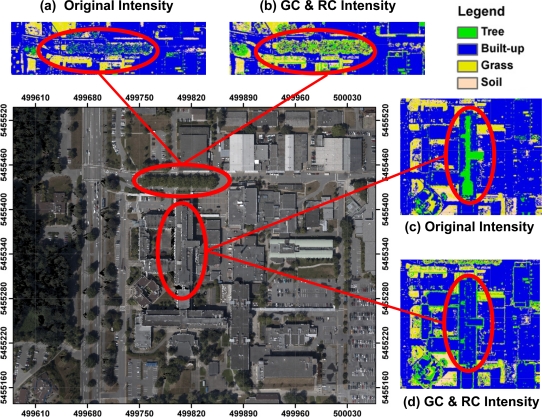
Comparison of classification results of original and the geometrically calibrated and radiometerically corrected LiDAR dataset.

Figure 6 shows the orthophoto used in the accuracy assessment process and the classification results of the intensity data before and after the geometric calibration and radiometric correction of the LiDAR data.

One can note a significant impact of the geometric calibration and radiometric correction in the tree class areas. In Figure 6a, the tree cluster along the main road is misclassified as built-up area using the original intensity data. However, the intensity values of tree clusters have significantly increased after radiometric correction. This leads to correct classification result as shown in Figure 6b since the difference of intensity values between built-up area and tree has increased after radiometric correction. In figure 6c, the roof top in the study area is entirely misclassified as tree in the result from the original intensity data. The results are improved after using the geometrically calibrated and radiometrically corrected intensity data (Figure 6d). Generally, the results show that the geometric calibration and radiometric correction of the airborne LiDAR data has a significant positive impact on the classification process, which would ultimately lead to better feature extraction and object recognition.

## 6. Conclusions and Recommendations for Future Work

In this research, methodologies for the geometric calibration and radiometric correction of the LiDAR system and collected data have been presented. The introduced geometric calibration procedure is denoted as the Quasi-rigorous due the fact that few reasonable assumptions are made for its development. This method only assumes that we are dealing with an almost vertical LiDAR system, which is quite realistic for flight missions with a steady platform. To conduct such a calibration, we require time-tagged point cloud and trajectory position data. In contrast to the position and orientation information requirement for each pulse in the rigorous calibration, the Quasi-rigorous procedure only requires a sample of the trajectory positions at a much lower rate. Access to this type of data is not a concern. Since this calibration procedure derives approximations of some of the system raw measurements, the proposed procedure can provide as a by-product the necessary information for the radiometric correction of the LiDAR intensity data when system raw measurements are not available (*i.e.*, improved scan mirror angles and ranges). It is important to mention that the introduced method is based on appropriate primitives (point-patch pairs) and do not require pre-processing of the LiDAR data. Moreover, the developed procedure provided a detailed analysis of the optimum flight and control configuration for reliable estimation of residual biases in the system parameters. The impact of the calibration process on the relative and absolute accuracy has been verified. The impact on the relative accuracy was evaluated by assessing the degree of compatibility between conjugate surface elements in overlapping strips before and after the geometric system calibration. The qualitative and quantitative assessments have demonstrated a significant improvement in the quality of fit between overlapping strips. The impact on the absolute accuracy was assessed by using the LiDAR data for photogrammetric georeferencing before and after performing the proposed geometric calibration procedure. The outcome of the photogrammetric reconstruction was evaluated through check point analysis. Significant improvement in the horizontal and vertical accuracy was demonstrated after removing the effect of estimated biases in the system parameters. Radiometric correction of the intensity data is also conducted on the geometrically calibrated LiDAR data to reduce the effects of atmospheric attenuation and scanning configuration. A physical model based on the radar equation is used for radiometric correction of the intensity data. The correction considers the system parameters, topographic effect, and atmospheric attenuation. After the radiometric correction, the homogeneity of the land cover classes is improved and the variance-to-mean ratio of the intensity data is reduced. It also enhances the separability amongst the different land cover classes. Finally, land cover classification is performed to investigate the impacts of the geometric calibration and radiometric correction on the classification process. A 7.5% accuracy improvement has been achieved in the classification results after conducting the proposed procedures.

Future work will focus on more testing using real datasets from operational systems. Also, the quasi-rigorous geometric calibration will be extended to include the attitude information in the calibration process. Furthermore, an automated procedure for the identification of useful areas within the data for reliable and faster estimation of the parameters will be implemented in the geometric calibration process. In addition, radiometric correction and land cover classification will be investigated by using full-waveform LiDAR data as it provides additional information (such as the transmitted laser pulse, the echo width, the cross section of the echo, etc.) comparing to the traditional multi-return LiDAR data. It is expected that the full-waveform LiDAR data will improve the point cloud density and the dimensionality of feature space leading to better classification and segmentation.

## Figures and Tables

**Table 1.** Experiments description (used overlapping strip-pairs and number of control points).

**Experiment**	**Overlapping Strip-Pairs**	**Number of Control Points**
I	1&2, 3&4, 4&5, 5&6	0
II	1&2, 3&4, 4&5, 5&6	37
III	1&2, 3&4, 4&5, 5&6	1
IV	1&2, 4&5, 5&6	37

**Table 2.** Estimated biases in the system parameters for the different experiments.

**Experiment**	***δ*Δ*X* (m)**	***δ*Δ*Y* (m)**	***δ*Δ*ω* (″)**	***δ*Δ*φ* (″)**	***δ*Δ*κ* (″)**	***δ*Δ*ρ* (m)**	***δS***
I	0.01	0.00	−29.5	−88.7	3.0	-	0.0002656
II	0.00	0.00	−29.0	−91.0	3.6	0.118	0.00010377
III	0.01	0.00	−29.5	−89.6	3.5	0.142	0.00009348
IV	0.02	0.05	−38.8	−90.7	−32.1	0.115	0.00003607

**Table 3.**
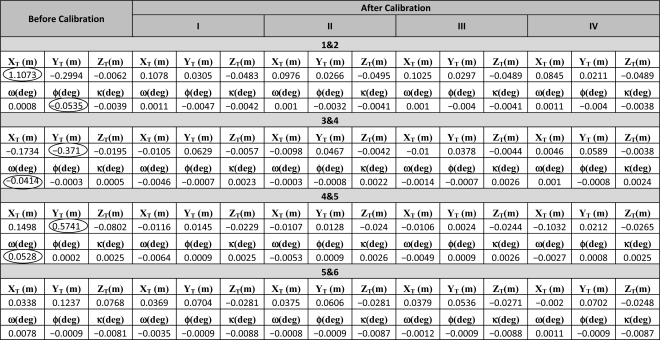
Discrepancies between overlapping strips before and after applying the calibration parameters estimated using the different scenarios.

**Table 4.**
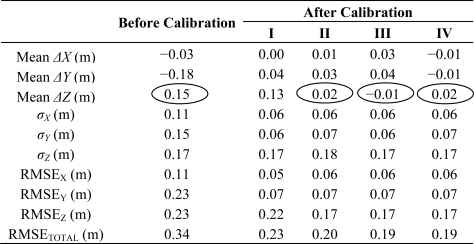
RMSE analysis of the photogrammetric check points using extracted control linear features from the LiDAR data before and after the calibration procedure.

**Table 5.** Mean and standard deviation of the intensity data for different land cover classes before and after radiometric correction.

	**Before Correction**	**After Correction**
Built-Up Area	13.9 ± 4.1	10.6 ± 3.3
Grassland	40.6 ± 9.5	32.7 ± 5.7
Soil	24.7 ± 5.1	20.5 ± 2.7
Tree	21.4 ± 9.3	82.7 ± 44.2

**Table 6.** Confusion matrix of the classification results using original LiDAR dataset.

	**Tree**	**Built-up**	**Grass**	**Soil**	**Total**	**KC**
Tree	130	40	20	18	208	0.464
Built-up	119	386	12	16	533	0.486
Grass	34	26	59	43	162	0.294
Soil	20	16	10	61	107	0.502

Total	303	468	101	138	1010	

Overall Accuracy = 63.0%			Average Kappa Coefficient (KC) = 0.442			

**Table 7.** Confusion matrix of the classification result using geometrically calibrated and radiometrically corrected LiDAR dataset.

	**Tree**	**Built-up**	**Grass**	**Soil**	**Total**	**KC**
Tree	153	23	12	4	192	0.725
Built-up	62	399	23	23	507	0.593
Grass	37	35	94	26	192	0.402
Soil	11	24	19	65	119	0.486

Total	263	481	148	118	1010	

Overall Accuracy = 70.4%			Average Kappa Coefficient (KC) = 0.558			

## Appendix A

### Modified Least Squares

1. Model

(A.1) $$y=\mathrm{Ax}+D+e\;\;\;e∼\left(0,\sum^{\prime }\right)\;\;\;\mathrm{where}\;\sum^{\prime }=\sigma_{o}^{2}P^{\prime +}\mathrm{and}\;P^{\prime }D=0$$

2. LSA Target Function ϕ(*x, D*)

(A.2) $$\phi \left(x,D\right)=e^{T}P^{\prime }e={\left(y-\mathrm{Ax}-D\right)}^{T}P^{\prime }\left(y-\mathrm{Ax}-D\right)=\min{|}_{x,D}$$

Since *P*^′^*D* = 0:

(A.3) $$\phi \left(x,D\right)=\phi \left(x\right)=e^{T}P^{\prime }e={\left(y-\mathrm{Ax}\right)}^{T}P^{\prime }\left(y-\mathrm{Ax}\right)=\min{|}_{x}$$

Expanding Equation (A.3) we get:

(A.4) $$\phi \left(x\right)={\left(y-\mathrm{Ax}\right)}^{T}P^{\prime }\left(y-\mathrm{Ax}\right)=y^{T}P^{\prime }y-y^{T}P^{\prime }\mathrm{Ax}-x^{T}A^{T}P^{\prime }y+x^{T}A^{T}P^{\prime }\mathrm{Ax}=\min{|}_{x}$$

Equation (A.3) can be simplified to:

(A.5) $$\phi \left(x\right)=y^{T}P^{\prime }y+x^{T}A^{T}P^{\prime }\mathrm{Ax}-2x^{T}A^{T}P^{\prime }y=\min{|}_{x}$$

3. Solution Vector *x̂*

The solution vector *x̂* that minimizes ϕ(*x*) can be obtained by differentiating ϕ(*x*) w.r.t. *x* and equating it to zero:

(A.6) $$\frac{\partial \phi }{\partial x}=2A^{T}P^{\prime }\mathrm{Ax}-2A^{T}P^{\prime }y=0$$

(A.7) $$\hat{x}={\left(A^{T}P^{\prime }A\right)}^{-1}A^{T}P^{\prime }y=N^{-1}A^{T}P^{\prime }y\;\;\;\;\;\mathrm{where}\;N=A^{T}P^{\prime }A$$

4. Variance-covariance matrix of the solution vector ∑{*x̂*}

Using the law of error propagation, the variance-covariance matrix of the solution vector ∑{*x̂*} can be obtained as follows:

(A.8) $$\sum \left\{\hat{x}\right\}=\sigma_{o}^{2}N^{-1}A^{T}P^{\prime }P^{\prime +}P^{\prime }AN^{-1}$$

Since for a pseudo inverse, *P*^′^*P*^′+^*P*^′^ = *P*^′^[32]

(A.9) $$\sum \left\{\hat{x}\right\}=\sigma_{o}^{2}N^{-1}NN^{-1}=\sigma_{o}^{2}N^{-1}$$

5. A-posteriori variance factor ${\hat{\sigma }}_{o}^{2}$

The a-posteriori variance factor ${\hat{\sigma }}_{o}^{2}$ is obtained by deriving the expected value of the sum squares of the weighted predicted residuals:

(A.10) $$E\left({\tilde{e}}^{T}P^{\prime }\tilde{e}\right)=E\left\{{\left(y-A\hat{x}-D\right)}^{T}P^{\prime }\left(y-A\hat{x}-D\right)\right\}$$

Since *P*^′^*D* = 0 Equation (A.10) gets the form:

(A.11) $$E\left({\tilde{e}}^{T}P^{\prime }\tilde{e}\right)=E\left\{{\left(y-A\hat{x}\right)}^{T}P^{\prime }\left(y-A\hat{x}\right)\right\}$$

Expanding Equation (A.11) while using the derived solution for *x̂* in Equation (A.7) we get:

(A.12) $$E\left({\tilde{e}}^{T}P^{\prime }\tilde{e}\right)=E\left\{y^{T}P^{\prime }y-y^{T}P^{\prime }AN^{-1}A^{T}P^{\prime }y\right\}$$

Given that the trace of a scalar equals to the scalar, i.e., tr(S) = S and that the trace operation is commutative, i.e., tr(AB) = tr(BA) [32], Equation (A.12) can be manipulated as follows:

(A.13) $$E\left({\tilde{e}}^{T}P^{\prime }\tilde{e}\right)=E\left\{\mathrm{tr}\left(P^{\prime }{\mathrm{yy}}^{T}\right)-\mathrm{tr}\left(P^{\prime }AN^{-1}A^{T}P^{\prime }{\mathrm{yy}}^{T}\right)\right\}$$

Based on the properties that tr(A) + tr(B) = tr(A+B) and that *E*{*tr*(*A*)} = *trE*(*A*) [32], Equation (A.13) can be rewritten as follows:

(A.14) $$E\left({\tilde{e}}^{T}P^{\prime }\tilde{e}\right)=\mathrm{tr}P^{\prime }\left[E\left({\mathrm{yy}}^{T}\right)-AN^{-1}A^{T}P^{\prime }E\left({\mathrm{yy}}^{T}\right)\right]=\mathrm{tr}P^{\prime }\left(I_{n}-AN^{-1}A^{T}P^{\prime }\right)E\left(yy^{T}\right)$$

where I_n_ is an *nxn* identity matrix.

The term *E*(*yy^T^*) can be derived from the variance-covariance matrix of the observations vector *∑*{*y*} as follows:

(A.15) $$\sum \left\{y\right\}=\sigma_{o}^{2}P^{\prime +}=E\left\{\left(y-\mathrm{Ax}-D\right)\;{\left(y-\mathrm{Ax}-D\right)}^{T}\right\}$$

Expanding Equation (A.15) we get:

(A.16) $$E({\mathrm{yy}}^{T})=\sigma_{o}^{2}P^{\prime +}+\left(\mathrm{Ax}+D\right)\;{\left(\mathrm{Ax}+D\right)}^{T}=\sigma_{o}^{2}P^{\prime +}+{\mathrm{Axx}}^{T}A^{T}+{\mathrm{AxD}}^{T}+{\mathrm{Dx}}^{T}A^{T}+{\mathrm{DD}}^{T}$$

Substituting Equation (A.16) in Equation (A.14) yields:

(A.17) $$E\left({\tilde{e}}^{T}P^{\prime }\tilde{e}\right)=\mathrm{tr}P^{\prime }\left(I-AN^{-1}A^{T}P^{\prime }\right)\left[\sigma_{o}^{2}P^{\prime +}+\mathrm{Ax}x^{T}A^{T}+\mathrm{Ax}D^{T}+Dx^{T}A^{T}+DD^{T}\right]$$

Given that *P*^′^*D =* 0, Equation (A.17) can be simplified to:

(A.18) $$E\left({\tilde{e}}^{T}P^{\prime }\tilde{e}\right)=\sigma_{o}^{2}\mathrm{tr}P^{\prime }\left(I-AN^{-1}A^{T}P^{\prime }\right)P^{\prime +}=\sigma_{o}^{2}\mathrm{tr}P^{\prime }P^{\prime +}-\sigma_{o}^{2}\mathrm{tr}N^{-1}A^{T}P^{\prime }P^{\prime +}P^{\prime }A$$

Based on the property that *tr*(*AB*) = *rank*(*AB*) (given that *AB* is idempotent) and *rank*(*AB*) ≤ min (*rankA, rankB*) [32], the following can be stated:

(A.19) $$\mathrm{tr}\left(P^{\prime }P^{\prime +}\right)=\mathrm{rank}\left(P^{\prime }P^{\prime +}\right)=\min\left(\mathrm{rank}P^{\prime },\mathrm{rank}P^{\prime +}\right)=\mathrm{rank}P^{\prime }=q$$

Given that *tr*(*P*^′^*P*^′+^) = *q* (as shown in Equation (A.19)) and that *P*^′^*P*^′+^*P*^′^ = *P*^′^, Equation (A.18) can be simplified to:

(A.20) $$E\left({\tilde{e}}^{T}P^{\prime }\tilde{e}\right)=\sigma_{o}^{2}q-\sigma_{o}^{2}\mathrm{tr}N^{-1}N=\sigma_{o}^{2}q-\sigma_{o}^{2}\mathrm{tr}I_{m}=\sigma_{o}^{2}q-\sigma_{o}^{2}m$$

where m is the number of unknown parameters.

Finally, we can get the expression for the a-posteriori variance factor $\sigma_{o}^{2}$ as follows:

(A.21) $${\hat{\sigma }}_{o}^{2}=\frac{E\left({\tilde{e}}^{T}P^{\prime }\tilde{e}\right)}{\left(q-m\right)}$$
